# Molecular-Genetic Research of *Rhodococcus rhodochrous* IEGM 1362, an Active (–)-Isopulegol Biotransformer

**DOI:** 10.3390/molecules30193976

**Published:** 2025-10-03

**Authors:** Polina Y. Maltseva, Natalia A. Plotnitskaya, Irina B. Ivshina

**Affiliations:** 1 Institute of Ecology and Genetics of Microorganisms of the Ural Branch of the Russian Academy of Sciences, Perm Federal Research Center of the Ural Branch of the Russian Academy of Sciences, 13 Golev Str., 614081 Perm, Russia; inbox.98@bk.ru (P.Y.M.); luchnikova.n@mail.ru (N.A.P.); 2 Department of Microbiology and Immunology, Perm State University, 15 Bukirev Str., 614990 Perm, Russia

**Keywords:** (–)-isopulegol, biotransformation, *Rhodococcus*, CYP450, qRT-PCR, gene expression, reference genes

## Abstract

The present study aimed to identify genes encoding enzymes involved in the biotransformation of monoterpenoid (–)-isopulegol by *Rhodococcus rhodochrous* IEGM 1362. This strain is able to transform (–)-isopulegol with formation of two novel metabolites with promising antitumor and analeptic activities. Cell fractions of rhodococci and specific inhibitor of cytochrome P450-dependent oxygenase activity were used to establish the localization and type of biotransformation enzymes. The expression of nine CYP450 genes selected by bioinformatics analysis was analyzed by quantitative real-time PCR (qRT-PCR). Selection of optimal reference genes for normalization of qRT-PCR results was performed using BestKeeper, Normfinder, geNorm, Delta CT, and RefFinder algorithms. As a result of these studies, the role of CYP450 enzyme complexes in the biotransformation of (–)-isopulegol was confirmed, and their cytoplasmic localization was established. The genes encoding DNA gyrase subunit B (*gyrB*) and protein translocase subunit A (*secA*) were selected as the most stable reference genes. The induced expression of the gene encoding CYP450 hydroxylase in the presence of (–)-isopulegol was determined. The obtained data allow us to identify the specific CYP450 enzyme involved in (–)-isopulegol biotransformation by *R*. *rhodochrous* IEGM 1362 and lay the foundation for further studies of molecular and genetic mechanisms of monoterpenoid biotransformation.

## 1. Introduction

Microbial transformation of terpene compounds has attracted increasing attention of researchers in recent years due to the prospect of obtaining new bioactive substances using environmentally safe methods. One of the actively developed substrates is (–)-isopulegol (C_10_H_18_O, CAS 89-79-2), a monoterpene alcohol widely used as a starting material for the synthesis of pharmaceuticals and fragrances due to its availability and low cost [1]. Despite the high industrial potential of (–)-isopulegol, information on its microbial transformation remains limited.

**Figure 1:**
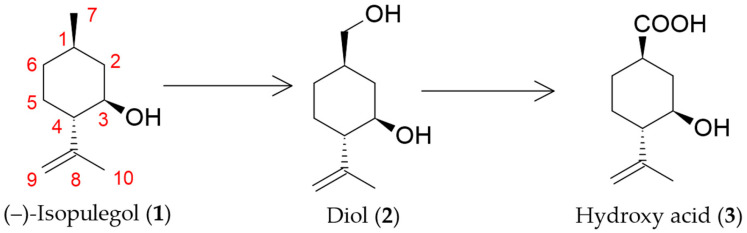
Biotransformation of (–)-isopulegol by *R*. *rhodochrous* IEGM 1362 [2].

In previous studies, we isolated a strain *R*. *rhodochrous* IEGM 1362 capable of transforming (–)-isopulegol (**1**) into two new compounds: (1*R*,2*S*,5*R*)-5-(hydroxymethyl)-2-(prop-1-en-2-yl)cyclohexanol (diol **2**) and (1*R*,3*R*,4*S*)-3-hydroxy-4-(prop-1-en-2-yl)cyclohexane carboxylic acid (hydroxy acid **3**) (Figure 1), for which antitumor and analeptic activities were predicted [2]. Detailed data on the chemical structure of the obtained compounds and the dynamics of the transformation process are presented in Supplementary Materials. Bioinformatics analysis of full-genome sequencing of *R*. *rhodochrous* IEGM 1362 identified 9 genes encoding CYP450 hydroxylases and oxygenases putatively associated with the metabolism of (–)-isopulegol [3]. Further studies of enzyme complexes and molecular and genetic mechanisms underlying this process are necessary for an in-depth understanding of (–)-isopulegol biotransformation.

Most of the known microbial transformations of terpenes, including hydroxylation, epoxidation, and oxidative degradation, are catalyzed by enzymes of the cytochrome P450 (CYP450) family [4]. CYP450 enzymes are heme-containing monooxygenases that catalyze a wide range of oxidative reactions with a high degree of chemo-, regio-, and stereoselectivity. Bacterial CYP450s are typically cytoplasmic enzymes and are often associated with the production of secondary metabolites. Although cytochromes involved in the bioconversion of the monoterpenoids 1,8-cineole [5], carveol [6], limonene-1,2-epoxide [7], *p*-cymene, limonene, and terpineol [8] have been extensively studied, there is a distinct lack of information on the regulation of (–)-isopulegol metabolism.

The aim of this work was to identify and analyze the expression of genes encoding key enzymes involved in the biotransformation of (–)-isopulegol by *R*. *rhodochrous* IEGM 1362. The results obtained will allow us to expand the understanding of the metabolic pathways of terpenes in actinomycetes and create a basis for the development of new biocatalytic methods for the production of valuable derivatives.

## 2. Results and Discussion

### 2.1. Determination of Key Enzymes of (–)-Isopulegol Biotransformation

Using cell fractions of *R*. *rhodochrous* IEGM 1362, it was revealed that the oxidation of (–)-isopulegol is provided by enzymes of cytoplasmic localization, as confirmed by the appearance of transformation products on thin-layer chromatography (TLC) (Figure S2). The detection of intracellular enzymatic activity indicates the likely involvement of cytoplasmic oxidoreductases and specific hydroxylases providing regio- and stereospecific oxidation of the substrate. Interestingly, the transformation of structurally more complex terpene compounds—triterpenoid betulin and diterpenoid dehydroabietinic acid—is catalyzed by enzyme complexes associated with the cell membrane [9,10]. Such a pattern may be due to the peculiarities of substrate distribution in the bacterial cell. Structurally complex hydrophobic compounds preferentially accumulate in membrane compartments where their interaction with the corresponding enzymatic systems takes place [11]. (–)-Isopulegol is a relatively small molecule with lower hydrophobicity compared to di- and triterpenoids and exhibits lower toxicity against actinomycetes, which probably accounts for its ability to penetrate the cytoplasm of rhodococci.

It should be noted that the used protocol has limitations and does not provide for complete separation or exclusion of contamination between cytoplasmic and membrane fractions. Thus, the localization of enzymes derived from these data is preliminary and indicative rather than definitive. Definitive confirmation of the precise intracellular localization of the enzymes will require application of more stringent subcellular fractionation techniques, such as ultracentrifugation. These analyses are planned as a part of future investigations.

**Figure 2:**
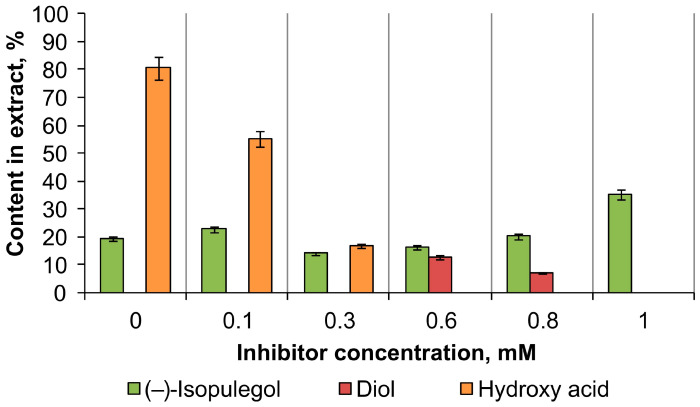
Changes in ethyl acetate extract composition (%) during biotransformation of (–)-isopulegol by *R*. *rhodochrous* IEGM 1362 with metyrapone.

According to GC-MS data, the introduction of the specific CYP450 inhibitor metyrapone into the biotransformation medium of (–)-isopulegol by *R*. *rhodochrous* IEGM 1362 cells significantly alters the kinetics of the process (Figure 2). At metyrapone concentrations of 0.1–0.3 mM, hydroxy acid predominates in the reaction products, but its yield decreases by 25–60% relative to the control without inhibitor. Increasing the concentration of metyrapone up to 0.8 mM leads to a decrease in substrate conversion and a decrease in the degree of oxidation of products, which is expressed in the accumulation of diol, probably due to the inhibition of key oxidoreductases. Complete suppression of (–)-isopulegol biotransformation is observed at 1 mM metyrapone concentration, which confirms the participation of cytochrome P450-dependent enzymes in the conversion of monoterpenoid.

Since metyrapone is not fully specific in bacteria and can affect other oxidoreductases, identifying the complete metabolic pathway, including other possible oxidoreductases involved, is the aim of our further research.

### 2.2. Selection of Reference Genes

Currently, quantitative real-time polymerase chain reaction (qRT-PCR) is recognized as the “gold standard” for assessing bacterial gene expression [12]. The most important step of this method is the selection of reference genes with stable expression levels under different culture conditions. Genes encoding enzymes involved in the maintenance of DNA structure and replication, transcription, translation, and regulation of protein metabolism are used as reference genes for rhodococci [13,14,15,16,17,18,19,20,21]. Despite the widespread use of so-called “housekeeping genes”, their stability needs to be confirmed experimentally for each specific research task [22]. The most frequently used reference gene is the gene encoding 16S ribosomal RNA. However, less than 25% of studies confirm its suitability, which is explained by the high variability of its expression under different experimental conditions [12].

Based on the literature data, we selected six candidate reference genes and matched them with specific primers for *R*. *rhodochrous* IEGM 1362 (Table S2) and optimal amplification conditions. Amplification of the candidate genes resulted in PCR products of the expected size for each primer pair, without formation of nonspecific products or primer dimers (Figure S3), and the melting curves for each primer pair were unimodal (Figure S4). The amplification efficiency of reference candidate genes ranged from 88.9% to 98.9%, and the correlation coefficients (R^2^) ranged from 0.914 to 0.998, meeting the requirements for qRT-PCR [23].

In the present study, the expression of six candidate genes for the role of reference genes (*16S rRNA*, *gyrA*, *ftsZ*, *gyrB*, *dnaG*, *secA*) was evaluated in order to select the most stable ones for normalization of qRT-PCR data. The cDNA of *R*. *rhodochrous* IEGM 1362 cells grown in four different conditions was used for analysis: (1) in RS medium; (2) in RS medium with (–)-isopulegol; in LB medium; and (4) in LB medium with (–)-isopulegol.

**Figure 3:**
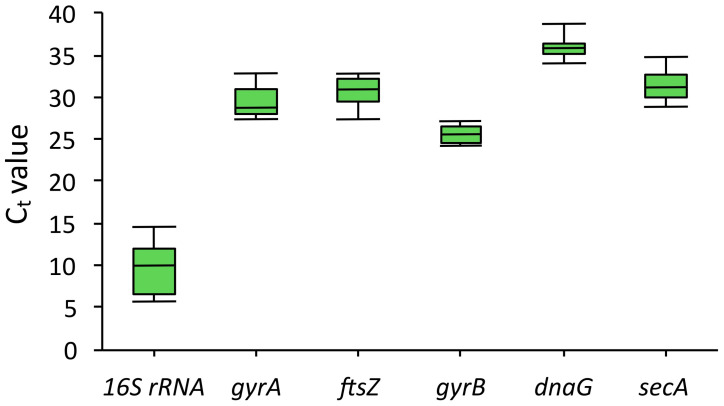
Distribution of C_t_ values for candidate reference genes. The line across the box depicts the median. The box indicates the 25th and 75th percentiles and the caps represent the maximum and minimum values.

Figure 3 shows the distribution of Ct (cycle threshold) values for each of the genes analyzed. Ct is defined as the number of PCR cycles required to achieve a fluorescence level that is significantly different from the background signal [12]. The Ct value is inversely correlated with the initial DNA concentration, which allows quantitative assessment of the target matrix content in the sample. The *16S rRNA* gene was characterized by the lowest Ct values (from 5 to 14), indicating its high expression in all the samples studied. However, such a high level of expression may result in reduced sensitivity when normalizing low-expressed genes such as cytochromes, limiting the possibility of using *16S rRNA* as a reference gene in this study. The *gyrA*, *ftsZ*, and *secA* genes showed similar expression levels, with Ct in the range of 27–34. At the same time, *gyrA* and *secA* showed greater variability in Ct values compared to *ftsZ*, which may indicate that their expression is less stable under different experimental conditions. The *dnaG* gene was characterized by the highest Ct values (from 34 to 38), indicating its extremely low expression level and potential instability when used as a reference gene. Of particular interest are the results obtained for the *gyrB* gene. Its Ct values ranged from 24 to 27, with minimal variability between samples. This combination of moderate expression level and high stability allows us to consider *gyrB* as an optimal candidate for normalization of qRT-PCR data.

**Figure 4:**
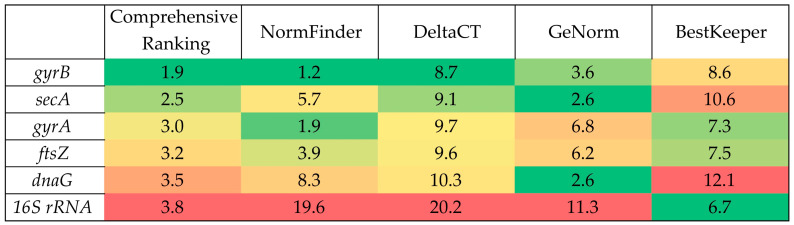
Heat map of expression stability of candidate genes for normalization of qRT-PCR data. Green corresponds to the highest expression stability, yellow corresponds to the average, and red corresponds to the lowest.

To select optimal reference genes, a comprehensive assessment of the stability of candidate gene expression based on Ct values was performed using five different algorithms: BestKeeper, Normfinder, geNorm, Delta CT, and RefFinder (Comprehensive ranking). The results were summarized in a single table presented as a heatmap (Figure 4). The numerical values in the cells reflect the stability indices obtained for each gene by the corresponding algorithm. The analysis of the obtained heat map showed that the *gyrB* gene has the highest stability when using most algorithms, which confirms its suitability as a reference gene for normalization of qRT-PCR data. The *secA* and *gyrA* genes can also be considered as alternative reference genes, since they occupy intermediate positions in terms of stability according to most algorithms. At the same time, *dnaG* and, especially, *16S rRNA* genes are characterized by the greatest variability of expression, which makes their use for normalization inappropriate.

Thus, based on the results of comprehensive evaluation, we selected *gyrB* and *secA* genes as optimal reference genes. In addition to high stability under the studied cultivation conditions, the combination of *gyrB* and *secA* provides coverage of two different functional categories (DNA replication and protein transport), which reduces the risk of coordinated regulation of these genes.

### 2.3. Expression Analysis of CYP450 Using qRT-PCR

To determine the contribution of CYP450 enzymes to the process of (–)-isopulegol conversion by *R*. *rhodochrous* IEGM 1362 cells, the expression of their encoding genes was studied. The CYP450 genes were previously identified by us in [3] (Table 1), and primers and optimal amplification conditions were selected for them. The melting curves for each primer pair were unimodal (Figure S5).

**Figure 5:**
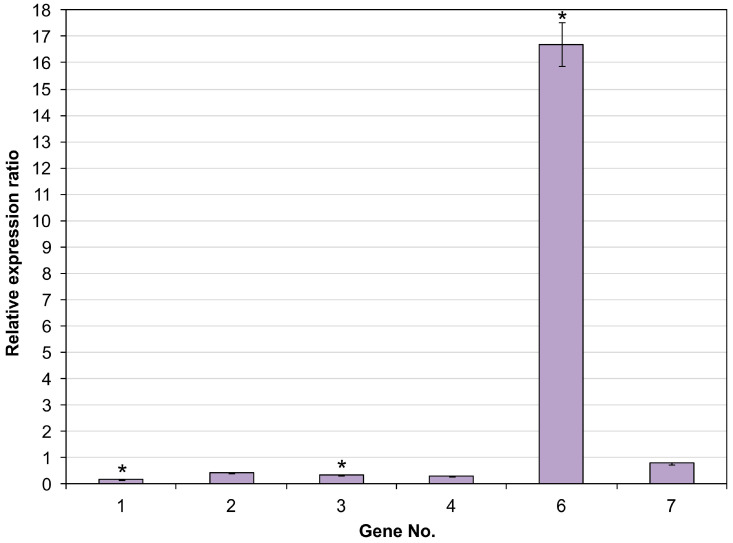
The relative CYP450 gene expression ratio (Pfaffl method) in *R*. *rhodochrous* IEGM 1362 in the presence of (–)-isopulegol. * The data are statistically reliable (*p*-value < 0.05).

To evaluate the role of the identified cytochrome P450 genes of *R*. *rhodochrous* IEGM 1362 in biotransformation of (–)-isopulegol, we analyzed the level of their expression in RS mineral-salt medium and in RS with addition of (–)-isopulegol. RS medium was chosen as the main one for expression analysis as it showed the most active biotransformation of (–)-isopulegol. Gene expression can vary significantly depending on the environmental composition, so we focused on conditions most relevant to the process under study. The *gyrB* and *secA* genes were used to normalize the results, and RS medium without monoterpenoid addition was used as a control. The relative expression ratio of CYP450 genes is shown in Figure 5; gene numbers correspond to Table 1. The table of calculations is presented in Supplementary Materials (Table S3).

Genes Nos. 2, 4 and 7 did not show significant difference in expression levels in all conditions examined, without marked induction by (–)-isopulegol. Expression of genes No. 1 and No. 3 were slightly reduced in the presence of monoterpenoid. This may indicate that these enzymes either have auxiliary functions in transformation or are involved in broader metabolic processes not specifically related to (–)-isopulegol.

Genes No. 5 and No. 8, despite the experimentally confirmed presence in the *R*. *rhodochrous* IEGM 1362 genome, were not expressed in any of the cultivation conditions. This may indicate that these genes are either activated under other growth conditions or represent pseudogenes or genes under the control of specific regulatory systems that were not activated in this experiment.

Expression of gene No. 9 was recorded only in the presence of (–)-isopulegol, which may indicate suppression of alternative metabolic pathways in the process of monoterpenoid transformation. This expression pattern indicates the possible involvement of this gene in the catabolism of other substrates that become less prioritized in the presence of (–)-isopulegol in the medium.

Gene No. 6 showed a high level of expression in the presence of (–)-isopulegol, 16 times higher than the control values, indicating its likely participation in the metabolism of this substrate. Such data strengthen the hypothesis that product of gene No. 6 encodes a key CYP450 responsible for the specific hydroxylation of (–)-isopulegol.

Previous bioinformatics analysis of the *R*. *rhodochrous* IEGM 1362 genome revealed a number of unique characteristics of gene No. 6, which indicate its likely role in the biotransformation of (–)-isopulegol [3]. Only gene No. 6 was not identified among the available whole-genome sequences of other strains of *R. rhodochrous*, while exhibiting high homology with a similar gene in *R. pyridinivorans* (up to 99.9% DNA and amino acid identity), which, along with the presence of a mobile element, suggests the possible horizontal transfer of this gene. Genetic environment No. 6, which includes the adjacent genes for ferredoxin and ferredoxin reductase, forms a probable functional cluster that is presumably responsible for the biotransformation of (−)-isopulegol, which may explain the specific catalytic activity of the strain. Furthermore, the 100% identity of gene No. 6 with one of the CYP450 genes of strain *R*. *rhodochrous* IEGM 107, an active transformer of mono- and di-terpenoids, highlights its role in the metabolism of terpenes by actinomycetes of the genus *Rhodococcus* [24].

**Figure 6:**
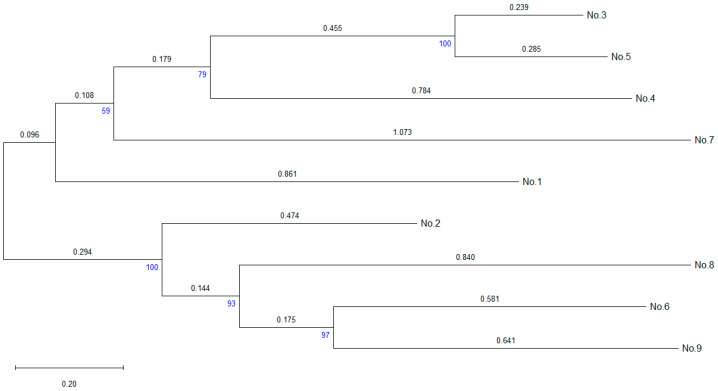
A distance tree for the pairwise alignments of the amino acid sequences of the CYP450 of *R. rhodochrous* IEGM 1362. The method used to construct the tree was Neighbour Joining. The bootstrap values (500 replicates) are shown below the branches.

To determine the evolutionary relationships between CYP450 enzymes, we constructed a phylogenetic tree based on amino acid sequences (Figure 6). The tree analysis showed a distinct structure with several cluster formations, reflecting differences in evolutionary divergence. Gene No. 1 is located at the base of the tree on the longest branch, indicating its significant evolutionary distance and uniqueness compared to the other CYP450s. Such an isolated position could indicate an ancient origin or horizontal gene transfer from another source, as well as a possible specialized function of the enzyme. Genes Nos. 6 and 9 form a compact cluster, indicating a high degree of homology and likely similarity in functional properties. A large cluster is formed by genes Nos. 3–5 and 7, exhibiting varying levels of divergence. Meanwhile, the short branches between genes Nos. 3, 4, and 5 indicate close homology and possibly similar substrate preferences. At the same time, longer branches leading to genes Nos. 2, 7, and 8 indicate deeper phylogenetic divergence and functional diversity.

Thus, the presented phylogenetic tree reveals the structural and functional diversity of CYP450 in *R*. *rhodochrous* IEGM 1362, which provides this strain with a wide range of biocatalytic capabilities.

The pairwise alignment of amino acid sequence of cytochrome No. 6 with known sequences of CYP450s from the UniProt database allowed us to identify homologous sequences with identity values up to 94.3% (Table 2). The identified homologous sequences with the highest degree of similarity corresponded to representatives of the genus *Rhodococcus*, as well as closely related species of *Nocardia* and *Gordonia*. Nevertheless, the identity level of cytochrome No. 6 with already identified cytochromes is not high, which emphasizes the novelty of our research and the relevance of further identification and characterization of CYP450 No. 6.

Thus, the experimental data obtained suggest that of the set of CYP450 genes of *R*. *rhodochrous* IEGM 1362, its gene No. 6 plays a key role in the biotransformation of (–)-isopulegol, since its expression is significantly and strictly induced by the presence of this substrate. Other genes likely have auxiliary functions in the metabolic process or are involved in the transformation of alternative compounds. These results expand the molecular understanding of the mechanisms of monoterpene biotransformation and provide a basis for further biotechnological optimization of the strain to improve the efficiency and selectivity of (–)-isopulegol transformations.

## 3. Materials and Methods

### 3.1. Strain and Culture Conditions

*R. rhodochrous* IEGM 1362 was isolated from the Paltinskoye peat deposit, Perm region, Russia, and deposited in the Regional Specialized Collection of Alkanotrophic Microorganisms (acronym IEGM, WFCC number 285). Along with (–)-isopulegol transformation, the strain also uses *n*-hexadecane as a sole carbon source, degrades dehydroabietic acid, resistant to Cr^6+^ (40.0 mM), Mo^6+^, and Zn^2+^ (5.0 mM), transforms monoterpenoids (–)-*cis*-carveol, (–)-*trans*-carveol, and (–)-L-carvone (http://www.iegmcol.ru/strains/rhodoc/rhodoch/r_rhod1362.html, accessed on 27 April 2025). The draft genome sequence data are available at the NCBI under GenBank accession number NZ_JANFQM000000000.1.

Culture was grown for 3 days in RS mineral salt medium (g/L: K_2_HPO_4_—2.0; KH_2_PO_4_—2.0; KNO_3_—1.0; (NH_4_)_2_SO_4_—2.0; NaCl—1.0; MgSO_4_—0.2; CaCl_2_—0.02, FeCl_3_ × 7H_2_O—0.001) or in a Luria–Bertani (LB) broth (Himedia, India), under shaking conditions (160 rpm) at 28 °C. In the RS medium, yeast extract (0.1 g/L), and trace element solution according to Postgate (0.1% *v/v*) were added. (–)-Isopulegol (Sigma-Aldrich, St. Louis, MO, USA) was used at a concentration of 0.025% *v/v*.

In some experiments, metyrapone, a CYP450 inhibitor, was used at concentrations 1.0 mM, 0.8 mM, 0.6 mM, 0.3 mM, and 0.1 mM.

The controls were: sterile RS medium with (–)-isopulegol (0.025% *v*/*v*) (abiotic control) and a culture of rhodococci grown in RS medium without (–)-isopulegol.

### 3.2. Cell Fractions Obtaining

Cells grown for 2 days in meat–peptone broth (MPB; Sigma-Aldrich, St. Louis, MO, USA) were washed three times and resuspended in phosphate-alkaline buffer (pH 7.0) and homogenized by ultrasound (Soniprep 150, MSE, Cholet, France, 10 μm, 45 min). The homogenate was centrifuged at 6000 rpm for 10 min (HERMLE Z200A, Hermle, Germany) to isolate cytoplasmic enzymes. The precipitate was resuspended in 1% Triton X-100 (Sigma-Aldrich, St. Louis, MO, USA) buffer, stirred on a shaker (30 min), and centrifuged to obtain the supernatant with membrane-bound enzymes. The remaining precipitate with firmly membrane-bound enzymes was resuspended in buffer.

### 3.3. Analytical Methods

Qualitative and quantitative analysis of the residual (–)-isopulegol and its derivatives were performed by thin-layer chromatography (TLC) and gas chromatography–mass spectrometry (GC-MS) as described in [2]. To detect the compounds, TLC plates were sprayed with 15% H_2_SO_4_ and heated at 100–120 °C for 2–3 min.

### 3.4. RNA Isolation and cDNA Synthesis

For RNA isolation, cells grown for 3 days were used. This time point was chosen according to analysis of respiratory activity and growth dynamics of *R*. *rhodochrous* IEGM 1362 (Figure S6), and correlated with the maximum catalytic activity of the cells toward (–)-isopulegol.

To isolate RNA, the cell sediment was washed from the medium with phosphate buffer (pH 7.0). 50 μL of lysozyme solution (40 mg/mL) was added to the precipitate and incubated for 1 h at 37 °C. Further RNA isolation was performed using the RNA Solo kit (Evrogen, Moscow, Russia) according to the manufacturer’s protocol. RNA concentration was assessed using a QubitTM fluorimeter (Thermo Fisher Scientific, Waltham, MA, USA). RNA purity was determined using a NanoPhotometer^®^ N50 (Implen, München, Germany). RT-PCR to obtain cDNA on RNA matrix was performed using a reagent kit with MMLV revertase (Evrogen, Moscow, Russia) according to the manufacturer’s instructions. Reaction mixture composition, μL: RNA matrix—6, random decanucleotide primer—3, 5x first strand synthesis buffer—4, dNTP—2, DTT—2, revertase—3, deionized and RNase-free water (Evrogen, Moscow, Russia).

### 3.5. Selection of Reference Genes

Primers were designed using Primer-BLAST service (https://www.ncbi.nlm.nih.gov/tools/primer-blast/index.cgi?LINK_LOC=BlastHome, accessed on 5 January 2025). The amplification efficiency (E) for each primer pair and the correlation coefficient (R^2^) were calculated using the calibration curve method. For this purpose, a series of tenfold dilutions of cDNA was prepared and the efficiency (E) was calculated using the formula:

$$E=(10^{\left(-\;\frac{1}{slope}\right)}-1)\times 100$$

The stability of reference gene candidates was evaluated using the RefFinder online service (https://www.ciidirsinaloa.com.mx/RefFinder-master/, accessed on 15 June 2025) [25], which includes four programs: geNorm, NormFinder, BestKeeper and the Delta CT method. All experiments were performed in three technical replicates.

### 3.6. Quantitative Real-Time PCR (qRT-PCR) and Gene Expression Analysis

The obtained cDNA was used for qRT-PCR with qPCRmix-HS SYBR (Evrogen, Russia, Russia) and primers selected using bioinformatics analysis [3] on a Real-time CFX Connect amplifier (Bio-Rad, Hercules, CA, USA). Reaction mixture composition, μL: cDNA—2, primers—1, 5x qPCRmix-HS—2, deionized and nuclease-free water—5. The PCR protocol included the following steps and conditions: 95.0 °C for 3 min, 40 cycles of 95 °C for 30 sec, 60 °C for 30 sec, 72.0 °C for 90 sec, a melting analysis of 0.5 °C increment from 65 to 95 °C (10 sec per cycle), and 72.0 °C for 10 min. Reaction specificity was assessed via melting curve analysis and 1.5% agarose gel electrophoresis for each primer pair. All experiments were performed in triplicate.

The controls were: (1) reaction mixture with cDNA and primers for *16S rRNA* (positive control); (2) reaction mixture without cDNA (negative control); (3) reaction mixture with RNA instead of cDNA (control of genomic DNA clearance).

The normalization of qRT-PCR results was performed with the reference genes selected in the current work, *gyrB* and *secA*. The relative gene expression was calculated using the Pfaffl method. If Ct values exceeded 35, they were considered background and indicated a lack of expression.

For the horizontal electrophoresis in an agarose gel (1.5% agarose in TBE buffer) Bio-Rad Gel Doc XR+ gel documentation system (Bio-Rad, Hercules, CA, USA) was used. Electrophoretic separation was carried out at a voltage of 70 V for 40 min. GelRed (Diaem, Moscow, Russia) was used as a nucleic acid dye. PCR products (5 µL) were added to an agarose gel in 4X Gel Loading Dye, Blue loading buffer (0.5 µL) (Evrogen, Moscow, Russia). To determine the size of the PCR products, a DNA length marker from 700 to 50 bp (Evrogen, Moscow, Russia) was added to the gel.

### 3.7. Bioinformatics Analysis

The pairwise alignment and phylogenetic tree construction of CYP450 gene products were performed using Clustal Omega (https://www.ebi.ac.uk/jdispatcher/msa/clustalo, accessed on 4 September 2025) [26] and MEGA12 [27]. Homologous amino acid sequences were searched using the pairwise alignment method with the UniProt online service, the BLASTP 2.16.0+ tool (https://www.uniprot.org/blast, accessed on 25 September 2025) [28].

## 4. Conclusions

In this study, the role of CYP450 enzymes in the biotransformation of (–)-isopulegol by *R*. *rhodochrous* IEGM 1362 was investigated. The complex approach, including the analysis of expression of nine CYP450 genes, determination of subcellular localization of enzymes and application of specific CYP450 inhibitor, allowed us to suggest the predominant cytoplasmic localization of these proteins and their direct participation in the metabolism of the monoterpenoid.

Of particular significance is the observed induction of expression of one of the genes encoding CYP450-hydroxylase in the presence of (–)-isopulegol. This emphasizes the important role of this enzyme in the main steps of biotransformation.

Further research will aim to clarify the role of CYP450 enzymes in biotransformation using CO-difference spectra for P450 content analysis and tests with alternative P450 inhibitors. The results obtained create prerequisites for further molecular genetics and biotechnological studies aimed at optimizing the monoterpenoid conversion process, including directed mutagenesis of functional genes and their heterologous expression.

## Figures and Tables

**Table 1:** Specific primers for genes encoding CYP450 enzymes of *R*. *rhodochrous* IEGM 1362 *.

Gene No.	Enzyme	Locus Tag	Primers (Forward; Reverse)	Amplicon Size, bp	PCR Efficiency, %	R^2^
1	Putative cytochrome P450 hydroxylase	NOT90_25380	TACAGCCCCGAACTCGACTA; TGTCCGGTATCGATGAAGCG	311	91.5	0.973
2	Putative cytochrome P450 hydroxylase	NOT90_21400	GTTCATCGAGGGCCTGAACA; CTCGAAACGCAGTGTCTCCT	373	93	0.999
3	Cytochrome P450	NOT90_15505	CGACGACATCTTCTCGGTGT; TGTGGTGGGAGAAGTGCATC	389	94.4	0.998
4	Putative cytochrome P450	NOT90_24995	TCGTTCGCTTTGCACTACCT; CGAATGGCTTGTATGCGTGG	370	99	0.999
5	Cytochrome P450	NOT90_03190	CACGTCGACCATCACGATCT; TTGGTGGACGATCGCTTTGA	448	100.2	0.999
6	Putative cytochrome P450 hydroxylase	NOT90_19635	GCTCTGACGCAGGAGTTCTT; ACGCAGTGTGTAGTCCTGTG	444	98.7	0.996
7	Cytochrome P450 monooxygenase	NOT90_21345	ATCGGTTCACCCAGAACCTG; GAGGACAGGATCACGAAGCC	403	100	0.966
8	Cytochrome P450	NOT90_02535	CCGATCTCGTAGCCCAGTTC; TTCGCTGATCTCGATTCCCG	554	96.9	0.997
9	Putative cytochrome P450 hydroxylase	NOT90_16895	CATCTCCCACGGCCTGTATC; TGTACTCGACACGAAGGTGC	461	102.9	0.993

* Modified from [3].

**Table 2:** The pairwise alignment of amino acid sequences of CYP450 No. 6 with known sequences of cytochromes P450 *.

Protein Name	Organism	Length, AA	Identity, %	Entry
Cytochrome P450	* Rhodococcus * sp. CX	440	94.3	A0A931HIH7
Cytochrome P450-terp	* Rhodococcus * sp. T7	440	92.3	A0A6N7CCE3
Cytochrome P450	* R. oxybenzonivorans *	440	91.1	A0A2S2C1W6
Cytochrome P450-terp	* Nocardia cerradoensis *	440	85.6	A0A231GXP1
Putative cytochrome P450	* Gordonia aichiensis * NBRC 108223	443	77.9	L7KKR0
Cytochrome P450	* G. asplenii *	442	77.2	A0A848KZE1
Cytochrome P450	* G. desulfuricans *	443	77	A0A7K3LNL8
Cytochrome P450-terp	* G. insulae *	441	76.8	A0A3G8JNI1
Cytochrome P450	* N. jinanensis *	443	76.5	A0A917RPB9
Cytochrome P450	* Amycolatopsis acidiphila *	441	71.2	A0A558AL67
Linalool 8-monooxygenase	* Candidatus Protofrankia datiscae *	441	70.6	F8AXG5

* Data is presented for sequences that have over 70% identity with the target protein.

## Data Availability

The data presented in this study are available in the Supplementary Materials and can be obtained on request from the corresponding author. The draft genome sequence data are available at NCBI under GenBank accession number NZ_JANFQM000000000.1.

## Supplementary Materials

The following supporting information can be downloaded at: https://www.mdpi.com/article/10.3390/molecules30193976/s1, Table S1: Changes in ethyl acetate extract (%) composition during biotransformation of (–)-isopulegol by *R*. *rhodochrous* IEGM 1362; Table S2: Specific primers for reference genes candidates of *R*. *rhodochrous* IEGM 1362; Table S3: Counting the relative expression ratio using Pfaffl method; Figure S1: Chromatogram of the extract obtained during biotransformation of (–)-isopulegol using *R*. *rhodochrous* IEGM 1362; Figure S2: TLC of (–)-isopulegol and its biotransformation products using cell fractions of *R*. *rhodochrous* IEGM 1362 cells; Figure S3: Electropherogram of PCR products of *R*. *rhodochrous* IEGM 1362 with specific primers for reference genes; Figure S4: Melting curve analysis of candidate reference genes; Figure S5: Melting curve analysis of CYP450 encoding genes; Figure S6: Oxygen consumption rate and growth dynamics of *R*. *rhodochrous* IEGM 1362.

## Abbreviations

The following abbreviations are used in this manuscript:

CYP450	Cytochrome P450
qRT-PCR	Quantitative real-time PCR
Ct	Cycle threshold
TLC	Thin-layer chromatography
GC-MS	Gas chromatography–mass spectrometry
MPB	Meat–peptone broth
LB	Luria–Bertani
