# Supplementary material for: Molecular-Genetic Research of Rhodococcus rhodochrous IEGM 1362, an Active (–)-Isopulegol Biotransformer
Source: Molecules. 2025 Oct 3;30(19):3976. doi: 10.3390/molecules30193976 (PMC12525638; doi:10.3390/molecules30193976)
Supplement: Supplementary file 1 [file molecules-30-03976-s001.zip › molecules-3907384-supplementary.pdf]

Biotransformation data

According to GC–MS, conversion of (−)-isopulegol (0.182 g) by *R. rhodochrous* IEGM 1362 was 90.2%, selectivity towards hydroxyl acid **3** was 25.7%; and for diol **2**, selectivity was 66.9%. Products **2** (0.017 g, 8.5%) and **3** (0.028 g, 12.9%) were isolated by column chromatography on SiO_2 [1]. Chromatogram of the reaction mixture and time-course data are presented at **Figure S1** and **Table S1**, respectively.

The structures of compounds **2** and **3** were confirmed by X-ray crystallography, which data was deposited at the Cambridge Crystallographic Data Centre (CCDC) with deposition numbers of CCDC 2168968 and 2168969, respectively. These data can be obtained free of charge from the CCDC via http://www.ccdc.cam.ac.uk/conts/retrieving.html, (or from the CCDC, 12 Union Road, Cambridge CB2 1EZ, UK; Fax: +44-1223-336033; E-mail: deposit@ccdc.cam.ac.uk).

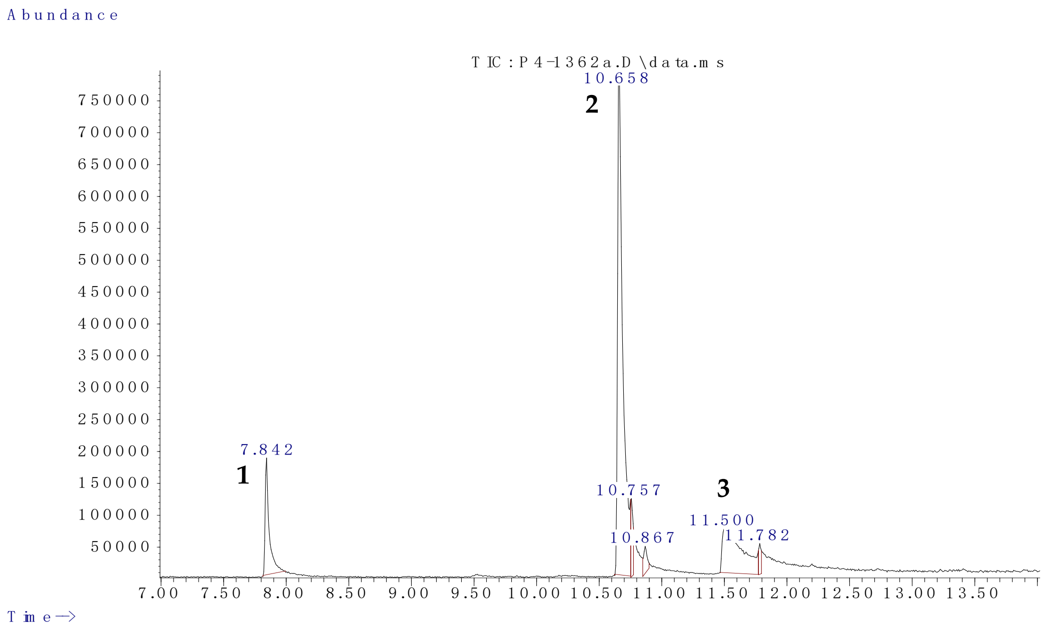

Figure S1. Chromatogram of the extract obtained during biotransformation of (−)-isopulegol using *R. rhodochrous* IEGM 1362: **1** – (−)-isopulegol; **2** – (1*R*,2*S*,5*R*)-5-(hydroxymethyl)-2-(prop-1-en-2-yl)cyclohexanol; **3** – (1*R*,3*R*,4*S*)-3-hydroxy-4-(prop-1-en-2-yl)cyclohexane carboxylic acid [1].

Table S1. Changes in ethyl acetate extract (%) composition during biotransformation of (−)-isopulegol by *R. rhodochrous* IEGM 1362*.

Days	(−)-Isopulegol 1	Diol 2	Hydroxy acid 3
Medium, 25 mL			
2	15.2	84.8	–
3	45.7	–	54.3
5	19.4	–	80.6
Medium, 100 mL			
2	–	–	100

*Modified from [1]

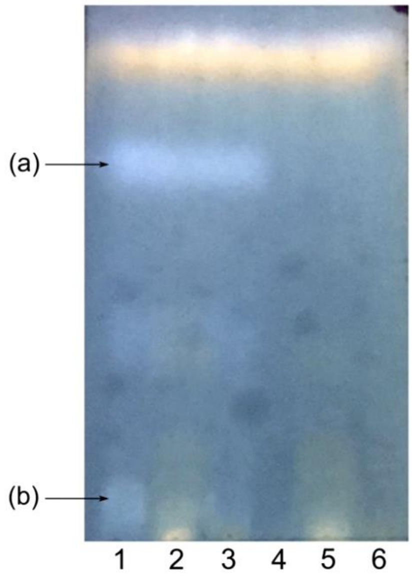

Figure S2. TLC of (–)-isopulegol (a) and its biotransformation products (b) using cell fractions of *R. rhodochrous* IEGM 1362 cells: 1, 4 – cytoplasmic enzymes; 2, 5 – extractable membrane-bound enzymes; 3, 6 – non-extractable membrane-bound enzymes; 4–6 – biotic control.

Table S2. Specific primers for reference genes candidates of *R. rhodochrous* IEGM 1362.

Gene	Gene annotation	Primers (forward; reverse)	Amplicon size, bp	PCR efficiency, %	R^2
16S rRNA	16S ribosomal RNA	GAGGGGTGGAAAGTTTTTCGGTGCAG GATGA; AGCCATGCACCACCTGTCTACCGG	839	98.9	0.995
gyrA	DNA gyrase subunit A	CGATGTTCGACAACGGCTTC; TGATGAGGTTGTCCGGGTTG	681	97.8	0.914
gyrB	DNA gyrase subunit B	GCGGCAAGATCATCAACGTC; TCCTTGGCGTTCATCTCACC	415	90.4	0.998
ftsZ	Cell division protein	GATCGAGCAGGGACTCAAGG; GTCGTTGGGGATGACGATGA	412	90.5	0.992
secA	Protein translocase subunit A	CACCGTCATCTACGAGGAGC; CCGACCGATTCCTCCTTCAG	513	97.8	0.994
dnaG	DNA primase	TACGATCCGCGACAACTACG; GTTCCATCACGAGCGAATGC	419	88.9	0.994

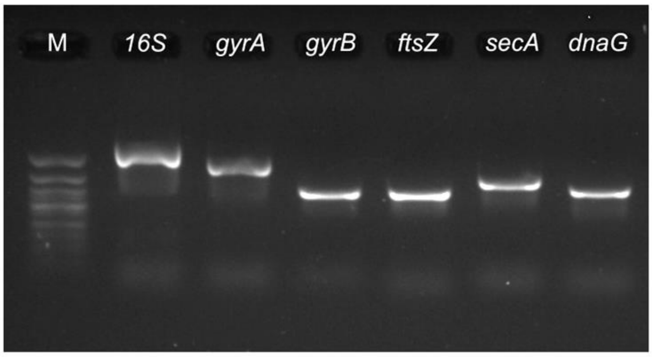

Figure S3. Electropherogram of PCR products of *R. rhodochrous* IEGM 1362 with specific primers for reference genes: M, DNA length marker from 700 to 50 bp.

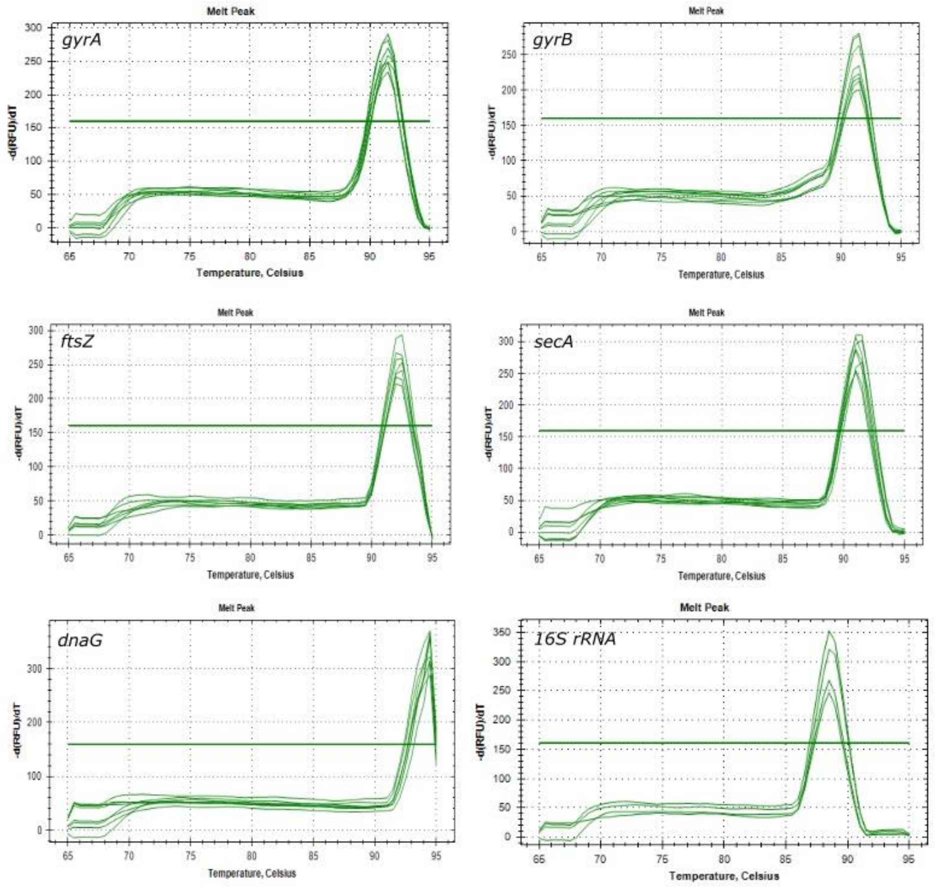

Figure S4. Melting curve analysis of candidate reference genes.

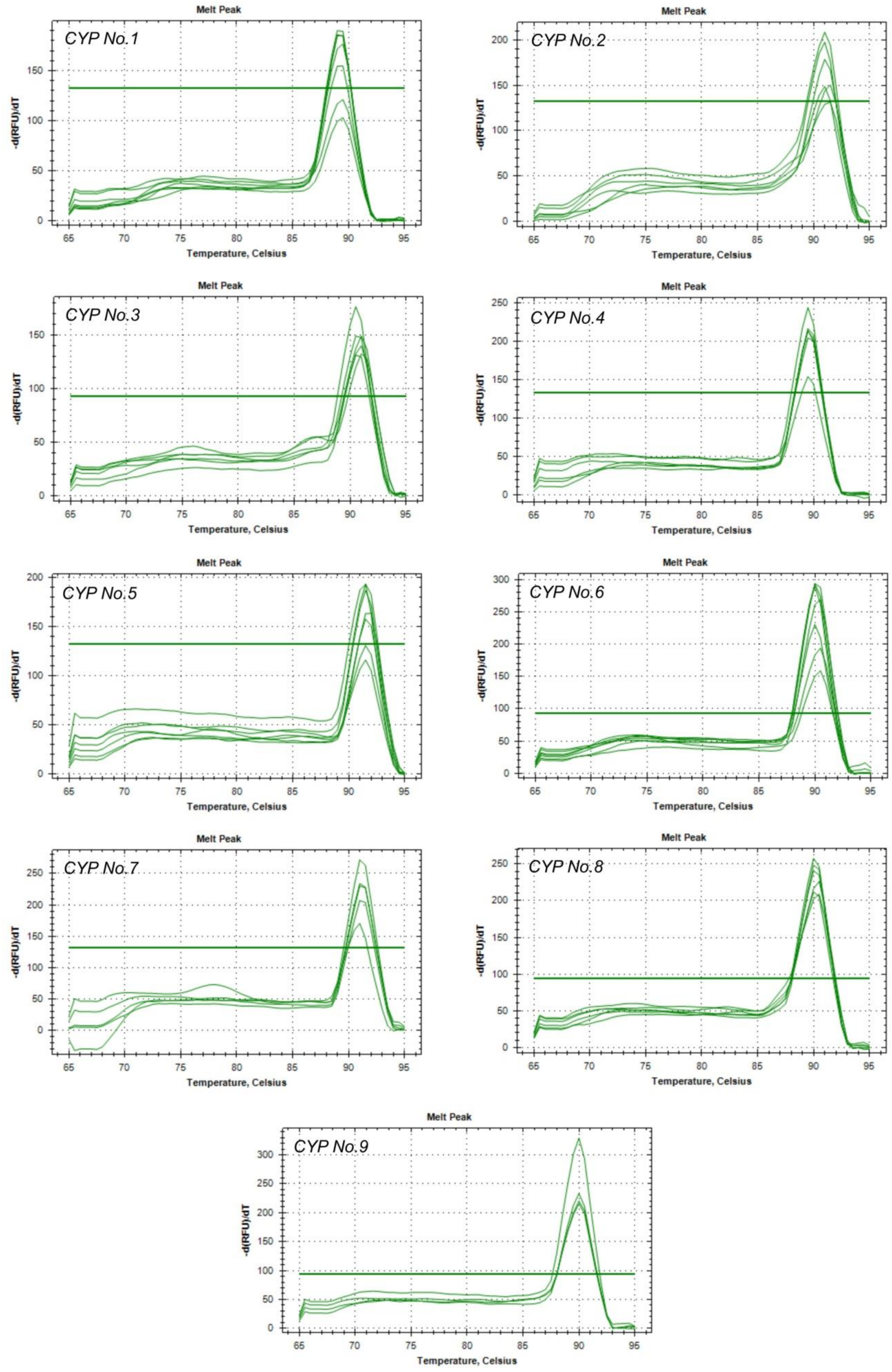

Figure S5. Melting curve analysis of CYP450 encoding genes.

Table S3. Counting the relative expression ratio using Pfaffl method (SD – standard deviation; CV – coefficient of variation; R – relative expression ratio).

Gene	Type	Ct mean (n = 3)	SD	CV, %	ΔCt	R
gyrB	control	29.21	1.01	3.44	0.43	–
	sample	28.78	0.27	0.93		
secA	control	32.94	1.04	3.16	2.30	–
	sample	30.64	0.31	1.02		
No. 1	control	29.21	0.94	3.22	-1.23	0.18*
	sample	30.44	0.17	0.56		
No. 2	control	33.58	0.68	2.04	0.03	0.41
	sample	33.55	1.44	4.28		
No. 3	control	32.65	0.37	1.14	-0.17	0.35*
	sample	32.82	0.64	1.95		
No. 4	control	33.92	0.12	0.35	-0.30	0.32
	sample	34.22	0.80	2.35		
No. 5	control	N/A	N/A	N/A	N/A	N/A
	sample	N/A	N/A	N/A		
No. 6	control	33.93	1.34	3.96	5.44	16.69*
	sample	28.48	0.18	0.62		
No. 7	control	34.06	0.91	2.68	0.98	0.79
	sample	33.08	1.21	3.67		
No. 8	control	N/A	N/A	N/A	N/A	N/A
	sample	N/A	N/A	N/A		
No. 9	control	34.08	0.74	2.17	N/A	N/A
	sample	N/A	N/A	N/A		

* The data are statistically reliable (p-value < 0.05).

N/A – the data are not available (no expression).

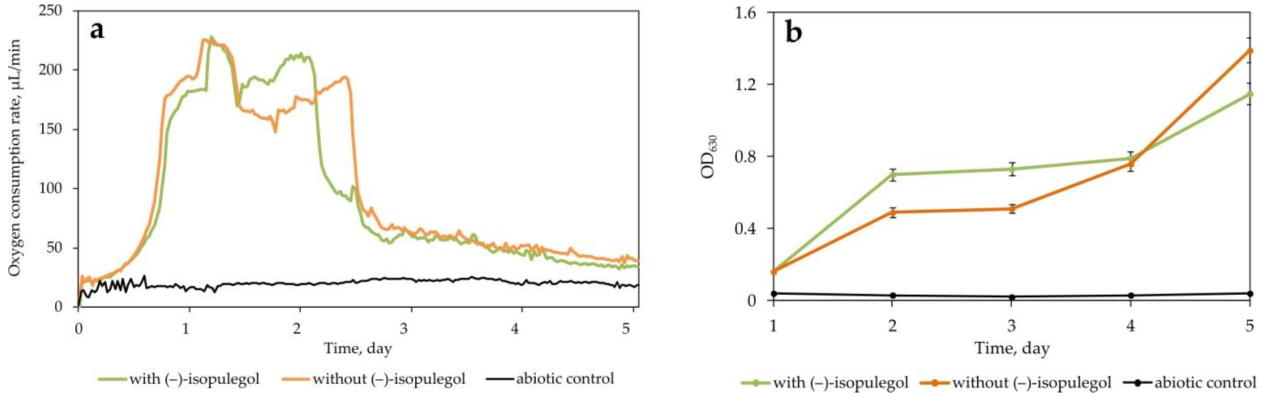

Figure S6. Oxygen consumption rate (**a**) and growth dynamics (**b**) of *R*. *rhodochrous* IEGM 1362*.
The culture was grown in 100 mL of RS medium with yeast extract (0.1 g/L), trace element solution according to Postgate (0.1% *v*/*v*) and (–)-isopulegol (0.025% *v*/*v*).
*Modified from [1]

References

1. Ivshina, I.B.; Luchnikova, N.A.; Maltseva, P.Y.; Ilyina, I.V.; Volcho, K.P.; Gatilov, Y.V.; Korchagina, D.V.; Kostrikina, N.A.; Sorokin, V.V.; Mulyukin, A.L. Biotransformation of (–)-Isopulegol by *Rhodococcus rhodochrous*. *Pharmaceuticals.* **2022**, *15*, 964.
